# Resilience among Tunisian adolescents during the COVID19 pandemic: about 326 cases

**DOI:** 10.1192/j.eurpsy.2024.1046

**Published:** 2024-08-27

**Authors:** K. Mayssa, B. Jaweher, K. Ali, K. Khaoula, B. T. Donia, H. Imen, A. Hela, M. Yousr

**Affiliations:** Department of Child Psychiatry, University Hospital of Hedi Chaker, Sfax, Tunisia

## Abstract

**Introduction:**

The COVID-19 pandemic has caused psychological distress in all the communities and through all ages. Some people seemed to be less affected and to be resilient because of a dynamic interaction between individual, relational and environmental factors.

**Objectives:**

We aim on this present study to evaluate the resilience and factors associated with it among a representative sample of Tunisian adolescents during the COVID19 pandemic.

**Methods:**

We conducted a cross sectional, descriptive and analytic study among Tunisian adolescents enrolled in secondary schools, in the Hamma region of the city of Gabes during the period extending from 5 March to 26 May 2021. Students were asked to complete a pre-established questionnaire, which contains questions about socio demographic features, medical history and knowledge about the pandemic of covid-19. The Child and Youth Resilience Measure (CYRM-28) was used to evaluate the global resilience and resources contributing to it. The CYRM-28 contains three resources including individual, relationship with primary caregivers and contextual factors. Higher scores reflect higher levels of factors associated with resilience.

**Results:**

A total of 326 adolescents aged between 14 and 18 years old participated on this study (mean age 16.65 years 1). There were 92 boys and 234 girls. In our sample, 4% of adolescents were infected by the Covid-19. The infection of a family member by this virus was noted in 27.3 % of cases. Adolescents were exposed to the death of a family member by Covid-19 in 22.4% of cases. The Global CYRM28 score was 105 ± 22.39. We found that adolescents who had loosen a family member because of the COVID infection, were less resilient than others (100 vs 107; p=0.023). Adolescents with a moderate to low socioeconomic level were less resilient especially with regard to contextual resilience (35, 57 vs 37, 83; p= 0.019). On the other hand, adolescents keeping means of leisure during this epidemic were significantly more resilient especially in the personal resources (p = 0.024).

**Conclusions:**

Our findings conclude to some individual, relational and contextual factors that contribute or alters the process of resilience. Recognizing the strengths and capacities of adolescents would allow the development of programs and resources that can help these young people develop resilience skills.

**Disclosure of Interest:**

None Declared

